# Heart Failure and Ischemic Stroke: A Bidirectional and Multivariable Mendelian Randomization Study

**DOI:** 10.3389/fgene.2021.771044

**Published:** 2021-11-29

**Authors:** Luyang Zhang, Weishi Liu, Wenxian Sun, Xin Wang, Mengke Tian, Lu-Lu Pei, Kai Liu, Jing Liang, Lue Zhou, Jie Lu, Mingming Ning, Ferdinando S. Buonanno, Yuming Xu, Bo Song

**Affiliations:** ^1^ Department of Neurology, The First Affiliated Hospital of Zhengzhou University, Zhengzhou, China; ^2^ Henan Key Laboratory of Cerebrovascular Diseases, Zhengzhou, China; ^3^ Clinical Systems Biology Laboratories, The First Affiliated Hospital of Zhengzhou University, Zhengzhou, China; ^4^ Department of Epidemiology and Health Statistics, College of Public Health, Zhengzhou University, Zhengzhou, China; ^5^ Clinical Proteomics Research Center and Cardio-Neurology Division, Massachusetts General Hospital, Harvard Medical School, Boston, MA, United States

**Keywords:** heart failure, ischemic stroke, single nucleotide polymorphism, mendelian randomization, stroke

## Abstract

**Background:** Heart failure (HF) is a potential cause of ischemic stroke (IS), and previous studies have reported an association between HF and IS. This study aimed to analyze the causal link between HF and IS using bidirectional and multivariable Mendelian randomization (MR) studies.

**Methods:** Genetic variants significantly associated with HF and IS were selected in the MR analysis from two large genome-wide association studies. Bidirectional and multivariable MR analyses were performed to evaluate the effect of HF on IS or the effect of IS on HF.

**Results:** Two-sample MR analysis showed causal effects of HF on IS of all causes [odds ratio (OR) = 1.555, 95% confidence interval (CI): 1.343–1.799, *p* = 3.35 × 10^−9^] and large artery atherosclerosis stroke (LAS) (OR = 1.678, 95% CI: 1.044–2.696, *p* = 3.03 × 10^−5^), while there was a suggestive effect of HF on cardioembolic stroke (CES) (OR = 3.355, 95% CI: 1.031–10.919, *p* = 0.044). Genetically predicted HF was not associated with small artery occlusion stroke. Bidirectional MR analysis showed causal effects of IS of all causes (OR = 1.211, 95% CI: 1.040–1.410, *p* = 0.014) and CES (OR = 1.277, 95% CI: 1.213–1.344, *p* = 6.73 × 10^−21^) on HF, while there were no causal effects of LAS on HF.

**Conclusion:** This MR analysis provided evidence of the causal links between genetically predicted HF and IS. Subgroup analysis highlighted the causal or suggestive relationship between genetically predicted HF and LAS or CES. The potential causal links need further investigation with genetic information about other ancestries or etiologies of HF.

## Introduction

Ischemic stroke (IS) is the leading cause of disability and mortality worldwide (Campbell et al., 2019). Because therapies aimed at treating IS are limited and are restricted by the time window or risk of bleeding, the early identification and control of high-risk factors of IS are of great importance (Prabhakaran et al., 2015; Phipps and Cronin, 2020).

Heart disease is one of the main causes of IS (Campbell et al., 2019) (Adams et al., 1993). It is recognized that atrial fibrillation (AF) contributes to cerebral embolism and IS with great severity (Kaarisalo et al., 1997). Additionally, heart failure (HF) is a potential cause of IS, and previous studies have reported an association between HF and IS (Haeusler et al., 2011). HF increases the risk of IS by nearly three times and accounts for 9% of IS cases (Kang et al., 2017). A population-based cohort study has demonstrated that patients with HF had 1.5- to 2.1- fold higher risk of IS compared to the general population during 31 days to 30 years of follow-up (Adelborg et al., 2017).

IS also induces cardiac complications (Chen et al., 2017). Status due to left ventricular systolic dysfunction, hypercoagulability, increased platelet aggregation, reduced fibrinolysis, endothelial dysfunction (Haeusler et al., 2011), and dynamic cerebral autoregulation (Caldas et al., 2017) are considered important mechanisms for the association between IS and HF. However, the causal links between IS and HF and the clinical benefits of HF treatments, such as diuretics, in patients with simultaneous IS and HF remain unclear. In addition, most studies have concentrated on the association between HF and cardioembolic stroke (CES). Nevertheless, approximately 70% of patients with small artery occlusion stroke (SAS) develop cardiac complications, and few studies have examined this phenomenon (McDermott et al., 1994; Chen et al., 2017).

Due to reverse causation and residual confounding, observational studies can rarely identify a causal link between IS and HF (Adelborg et al., 2017; Iguchi et al., 2021). In addition, HF and IS share some risk factors, like hypertension, diabetes mellitus, hyperlipidemia, obesity, and smoking history (Haeusler et al., 2011). This may hinder the identification of the association between HF and IS or lead to its underestimation. Mendelian randomization (MR), which utilizes genetic variations as instrumental variables (IVs), is a tool for analyzing the causal link between exposures and outcomes (Lawlor et al., 2008; Sekula et al., 2016). MR can be considered a natural randomized controlled trial for the assumption that the exposure-related genetic variants are randomly allocated at conception (Emdin et al., 2017). Therefore, MR can overcome confounding and reverse causation in the absence of pleiotropy, which are limitations of observational studies, and it is suitable for analyzing the relationship between IS and HF (Latvala and Ollikainen, 2016).

Our study aimed to analyze the causal link between HF and IS and its subtypes [large artery atherosclerosis stroke (LAS), CES, and SAS] *via* bidirectional and multivariable MR analyses.

## Materials and Methods

### Data Sources

Genetic variants significantly associated with HF were extracted from a large genome-wide association study (GWAS) by Regeneron Genetics Center, which comprised 47,309 cases and 930,014 controls of European ancestry (Shah et al., 2020). We obtained the genetic variants associated with IS and its subtypes from a large GWAS database constructed by MEGASTROKE, which comprised 34,217 cases and 406,111 controls, including LAS (4,373 cases), CES (7,193 cases), and SAS (5,386 cases) (Malik et al., 2018). The corresponding single nucleotide polymorphisms (SNPs) were obtained from the HF and IS datasets.

Other GWAS datasets obtained in our study included: body mass index (BMI) with 315,347 subjects from Hoffmann et al.; systolic and diastolic blood pressure (SBP and DBP) with 757,601 subjects from the International Consortium of Blood Pressure; fasting blood glucose (FBG) with 58,074 subjects from Manning et al.; glycosylated hemoglobin (HbA1c) with 9,436 subjects from Prins et al.; total cholesterol (TC), low-density lipoprotein cholesterol (LDL), and triglyceride (TG) with 187,365, 173,082, and 177,861 subjects, respectively, from the Global Lipids Genetics Consortium; apolipoprotein A-1 (ApoA1) with 393,193 subjects from United Kingdom Biobank; cigarettes smoked per day with 68,028 subjects from the Tobacco and Genetics Consortium; alcohol consumption with 112,117 subjects from Clarke et al.; AF with 1,036,836 subjects from Nielsen et al.; and coronary heart disease (CHD) with 547,261 subjects from van der Harst et al. (Manning et al., 2012; Willer et al., 2013; Clarke et al., 2017; Prins et al., 2017; Evangelou et al., 2018; Hoffmann et al., 2018; Nielsen et al., 2018; van der Harst and Verweij, 2018; Richardson et al., 2020) (Supplementary Table S3).

### SNP Selection Criteria

SNPs significantly associated with HF or IS (*p* < 5 × 10^−8^) were selected as IVs from the GWAS datasets of HF or IS, respectively. Then, linkage disequilibrium of the SNPs was tested, and SNPs with *r* ^2^ > 0.01 in the European samples of 1,000 genomes were excluded. *R* ^*2*^, the proportion of variance in the exposure explained by the specific genetic variant, was calculated by the formula: *R* ^*2*^ = 2 × β^2^ × EAF × (1−EAF), where β represented the estimated effect of the genetic variant and EAF indicated effect allele frequency. F-statistic was further calculated by the formula: F = *R* ^*2*^ × (*N*–*k*−1)/*k* (1−*R* ^*2*^), where *N* represented the sample size and *k* represented the number of included genetic variants. Genetic variants with F-statistics < 10 were considered weak IVs and were excluded in the MR analysis. Twelve SNPs associated with HF at genome-wide significance were first identified, and one weak genetic variant (rs4135240) was excluded (Table 1). Regarding IVs for IS, 12, 9, 4, and 4 SNPs remained for HF, IS, LAS, and CES, respectively (Supplementary Table S1). A proxy SNP in linkage disequilibrium (*r* ^2^ > 0.9) was included if a certain SNP was unavailable in the outcome data.

**TABLE 1:** Included SNPs that are significantly associated with heart failure.

SNP	Nearby gene	Ch.	EA	OA	EAF	HF				
						β	SE	*p*-value	*R* ^2^	F statistic
rs660240	CELSR2	1	C	T	0.79	0.0611	0.0097	3.25 × 10^−10^	0.0012	101
rs17042102	PITX2, FAM241A	4	A	G	0.12	0.1103	0.0121	5.71 × 10^−20^	0.0026	210
rs11745324	KLHL3	5	A	G	0.77	−0.0528	0.0095	2.34 × 10^−8^	0.0010	81
rs1510226	SLC22A3	6	C	T	0.66	0.1620	0.0285	1.27 × 10^−8^	0.0118	971
rs55730499	LPA	6	T	C	0.07	0.1058	0.0157	1.83 × 10^−11^	0.0015	119
rs4135240	CDKN1A, DINOL	6	C	T	0.02	−0.0486	0.0084	6.84 × 10^−9^	0.0001	8
rs600038	ABO	9	C	T	0.21	0.0569	0.0096	3.68 × 10^−9^	0.0011	88
rs1556516	CDKN2B-AS1	9	C	G	0.48	0.0622	0.0078	1.57 × 10^−15^	0.0019	158
rs4746140	SYNPO2L, AGAP5	10	C	G	0.85	−0.0666	0.0109	1.10 × 10^−9^	0.0011	92
rs17617337	BAG3	10	T	C	0.78	−0.0561	0.0095	3.65 × 10^−9^	0.0011	88
rs4766578	ATXN2	12	A	T	0.47	−0.0433	0.0079	4.90 × 10^−8^	0.0009	76
rs56094641	FTO	16	G	A	0.42	0.0454	0.0080	1.21 × 10^−8^	0.0010	82

Ch., chromosome; EA, effect allele; EAF, effect allele frequency; HF, heart failure; OA, other allele; SE, standard error; SNP, single nucleotide polymorphism.

### Statistical Analysis

**FIGURE 1:**
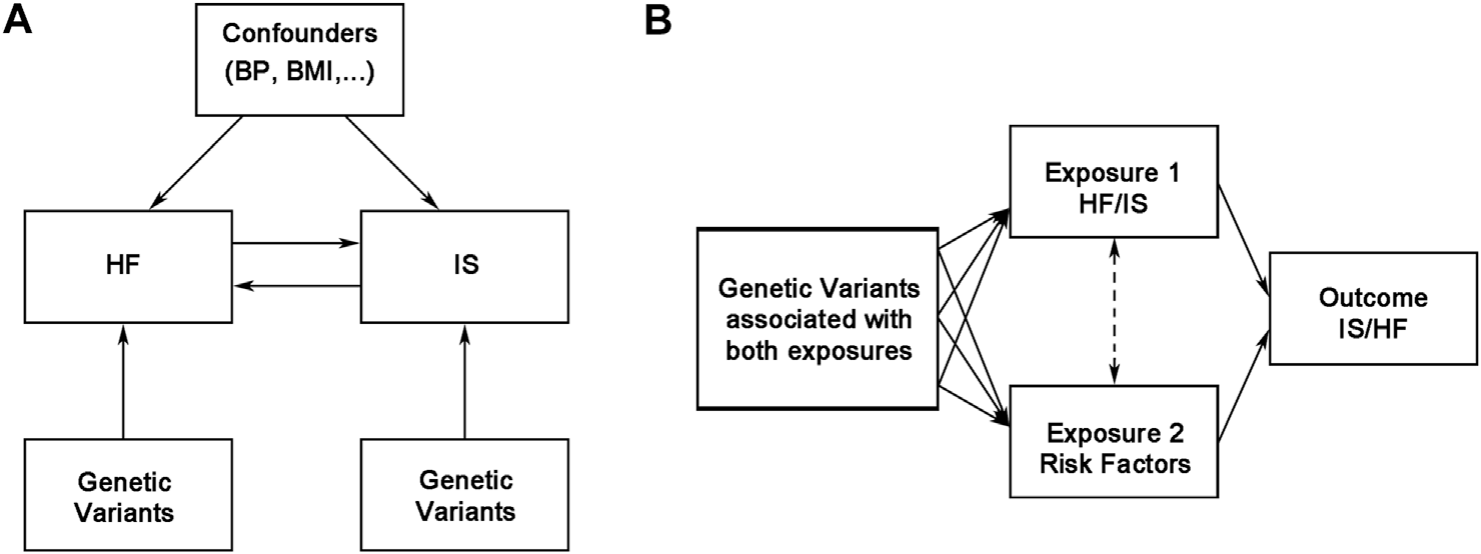
Overview of the bidirectional and multivariable Mendelian randomization study design. BMI, body mass index; BP, blood pressure; HF, heart failure; IS, ischemic stroke.

The MR study was performed in R version 4.0.2 (The R Development Core Team, Vienna, Austria) using the *TwoSampleMR* R package version 0.5.5 and MRInstruments version 0.3.2R package, curated by MR-Base (http://www.mrbase.org) (Hemani et al., 2018). Two-sample MR and multivariable MR analyses were performed through the function *mr* and *mv_multiple*, respectively (Hemani et al., 2018). LDLink (version 5.0) was used to test for linkage disequilibrium. The design of our MR analysis is shown in Figure 1, and our MR study was based on three assumptions. First, the IVs were significantly associated with exposures. Second, the IVs were not associated with confounding factors. Third, the IVs were relevant to the outcome only via exposure (Figure 1) (Emdin et al., 2017).

For MR analysis, we estimated the causal link between exposures and outcomes by an inverse variance-weighted (IVW) method (Hemani et al., 2018). We also used the simple median, simple mode, weighted mode, robust adjusted profile score (RAPS), Bayesian Weighted Mendelian Randomization (BWMR), and Mendelian randomization pleiotropy residual sum and outlier (MR-PRESSO) methods for additional analysis (Bowden et al., 2016; Hartwig et al., 2017; Verbanck et al., 2018; Zhao et al., 2020). Sensitivity analysis included a heterogeneity test (Cochrane’s Q test), pleiotropy test (MR-Egger intercept test), and leave-one-out analysis (Bowden et al., 2015). Once heterogeneity was identified (*p* < 0.05), the multiplicative random effects IVW method was used for estimating causal effect. A Bonferroni correction (*p*-value after correction = 0.05/*X*/*Y*, *X* indicating the number of exposures and *Y* indicating the number of outcomes) was used for multiple comparisons. A *p*-value between corrected *p*-value and 0.05 was defined as a suggestive association between the exposure and the outcome. For multivariable analysis, we included any single cardiovascular risk factor (including BMI, SBP, DBP, FBG, HbA1c, TC, LDL, TG, ApoA1, cigarettes smoked per day, alcohol consumption, AF, and CHD) in the analysis for adjustment. The IVW method was used for the causal estimates in the multivariable analysis.

## Results

### Effects of Genetically Predicted HF on IS

**FIGURE 2:**
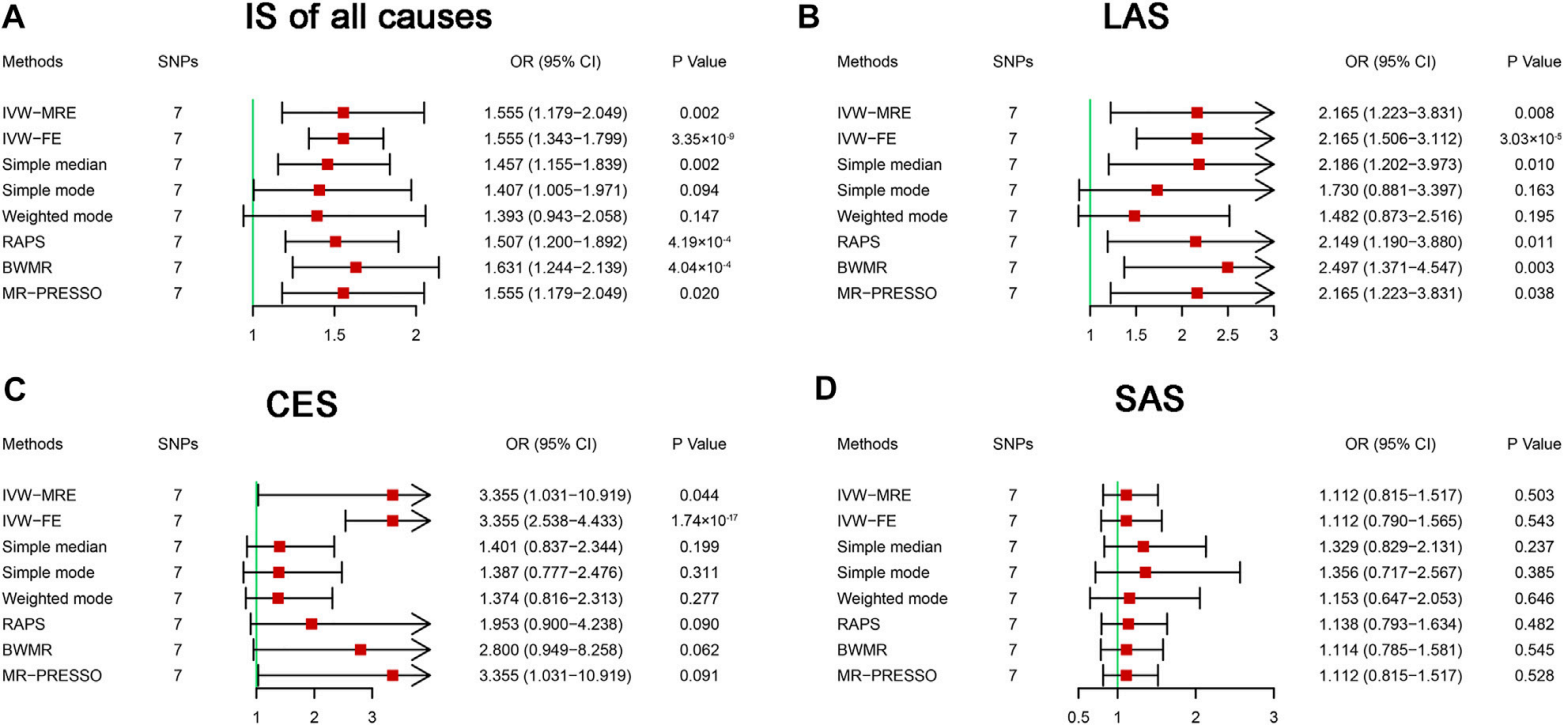
Mendelian randomization analysis of the effect of heart failure on ischemic stroke. BWMR, Bayesian weighted Mendelian randomization; CES, cardioembolic stroke; CI, confidence interval; IS, ischemic stroke; IVW-FE, inverse variance-weighted-fixed effects; IVW-MRE, inverse variance-weighted-multiplicative random effects; LAS, large artery atherosclerosis stroke; MR-PRESSO, Mendelian randomization pleiotropy residual sum and outlier; OR, odds ratio; RAPS, robust adjusted profile score; SAS, small artery occlusion stroke; SNP, single nucleotide polymorphism. Bonferroni corrected *p*-value = 0.0125.

After evaluating linkage disequilibrium and removing weak SNPs, 11 SNPs associated with HF were included in the MR analysis of HF and IS (Table 1 and Supplementary Table S1). The main results are shown in Figure 2. IVW (fixed effects) analysis indicated causal links between HF and IS of all causes [odds ratio (OR) = 1.555, 95% confidence interval (CI): 1.343–1.799, *p* = 3.35 × 10^−9^], LAS (OR = 1.678, 95% CI: 1.044–2.696, *p* = 3.03 × 10^−5^), and CES (OR = 3.355, 95% CI: 2.538–4.433, *p* = 1.74 × 10^−17^), but no causal links between HF and SAS were identified (OR = 1.112, 95% CI: 0.790–1.565, *p* = 0.543). Sensitivity analysis indicated the existence of heterogeneities in the analysis between HF and IS of all causes (*p* = 0.037), LAS (*p* = 0.014), and CES (*p* = 5.81 × 10^−15^) (Table 2). The multiplicative random effects IVW method was further used and indicated causal effects of HF on IS of all causes (OR = 1.555, 95% CI: 1.179–2.049, *p* = 0.002), LAS (OR = 2.165, 95% CI: 1.223–3.831, *p* = 0.008), and CES (OR = 3.355, 95% CI: 1.031–10.919, *p* = 0.044) (Figure 2). After multiple comparisons (corrected *p*-value = 0.05/1/4 = 0.0125), the causal effect of HF on CES was not robust (Figure 2). No pleiotropy was identified by the MR-Egger intercept analysis (Table 2). Leave-one-out analysis showed that the links between HF and IS, LAS, CES, and SAS were not substantially driven by any individual SNP (Supplementary Figure S1).

**TABLE 2:** Sensitivity analysis of heart failure and ischemic stroke.

	Pleiotropy		Heterogeneity	
	Intercept	*p*-value	*Q*	*p*-value
HF-IS				
Outcomes				
IS	−0.044	0.100	11.830	0.037
LAS	−0.025	0.693	14.344	0.014
SAS	0.034	0.354	3.903	0.563
CES	−0.153	0.209	75.980	5.81 × 10^−15^
IS-HF				
Exposures				
IS	−0.052	0.329	15.566	0.016
LAS	0.017	0.538	1.153	0.283
CES	−0.008	0.783	3.317	0.069

CES, cardioembolic stroke; HF, heart failure; IS, ischemic stroke; LAS, large artery atherosclerosis stroke; SAS, small artery occlusion stroke.

**FIGURE 3:**
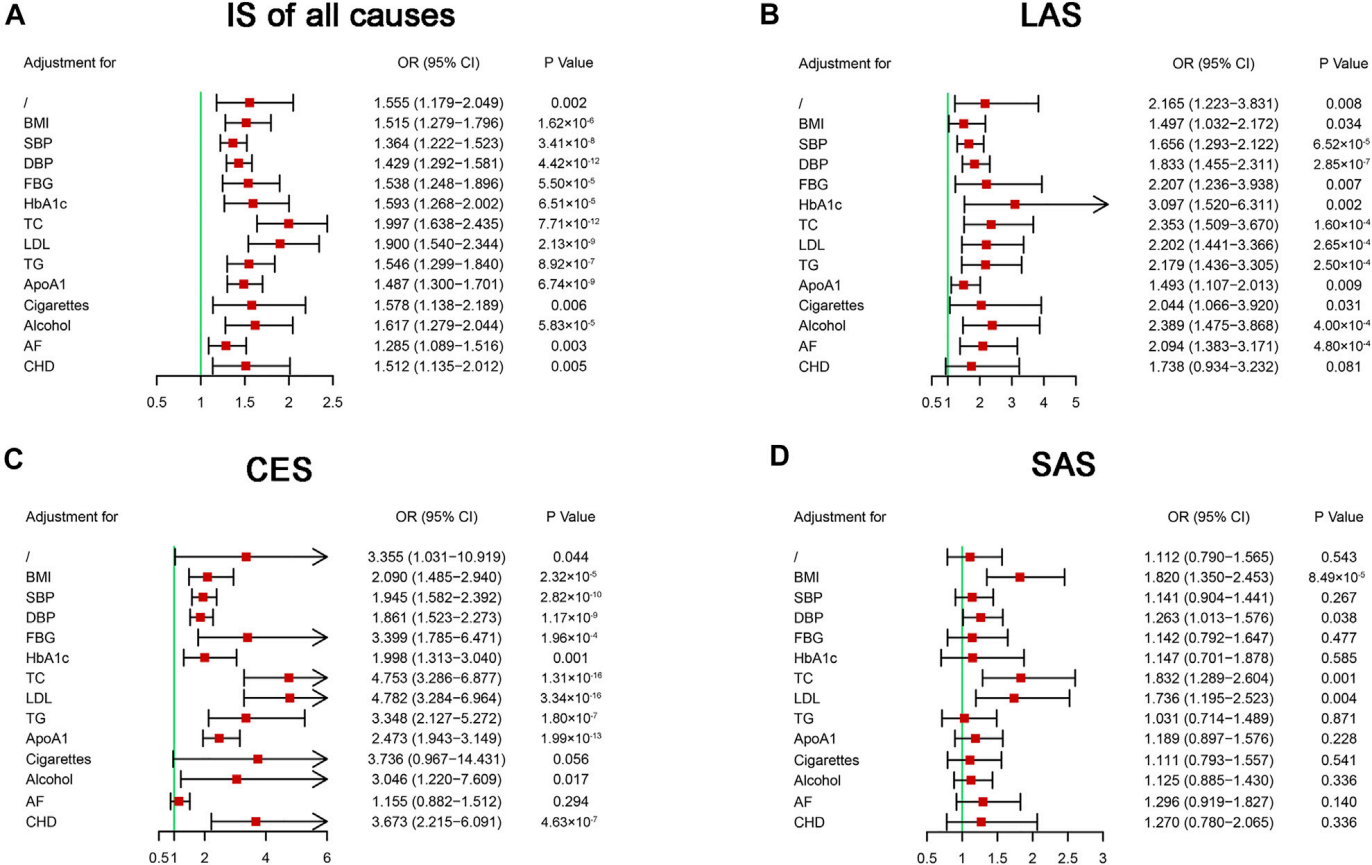
Multivariable Mendelian randomization analysis of the effect of heart failure on ischemic stroke. ApoA1, apolipoprotein A-1; AF, atrial fibrillation; BMI, body mass index; CES, cardioembolic stroke; CHD, coronary heart disease; CI, confidential interval; DBP, diastolic blood pressure; FBG, fasting blood glucose; HbA1c, glycosylated hemoglobin; IS, ischemic stroke; LAS, large artery atherosclerosis stroke; LDL, low-density lipoprotein cholesterol; OR, odds ratio; SAS, small artery occlusion stroke; SBP, systolic blood pressure; SNP, single nucleotide polymorphism; TC, total cholesterol; TG, triglyceride.

To further evaluate the causal effects of HF on IS and its subtypes, we performed multivariable MR analysis to avoid pleiotropy (Burgess and Thompson, 2015). Our analysis indicated that no single vascular risk factor could adjust the causal effect of HF on IS of all causes (Figure 3A). After including CHD as a second exposure, the effect of HF on LAS was not statistically significant (OR = 1.738, 95% CI: 0.934–3.232, *p* = 0.081) (Figure 3B). The causal effect of HF on CES was not significant after including AF in the exposures (OR = 1.155, 95% CI: 0.882–1.512, *p* = 0.294) (Figure 3C). The causal effect of HF on SAS was still significant after adding some vascular risk factors (BMI, DBP, TC, and LDL) in the exposures (Figure 3D). On MR analysis, we found a causal effect of HF on IS of all causes that one unit increase in the genetically predicted odds of HF could increase the risk of IS of all causes by approximately 55.5%. In the subgroups of IS, HF had a causal effect on LAS, a suggestive effect on CES, and no causal effect on SAS.

### Effects of Genetically Predicted IS on HF

**FIGURE 4:**
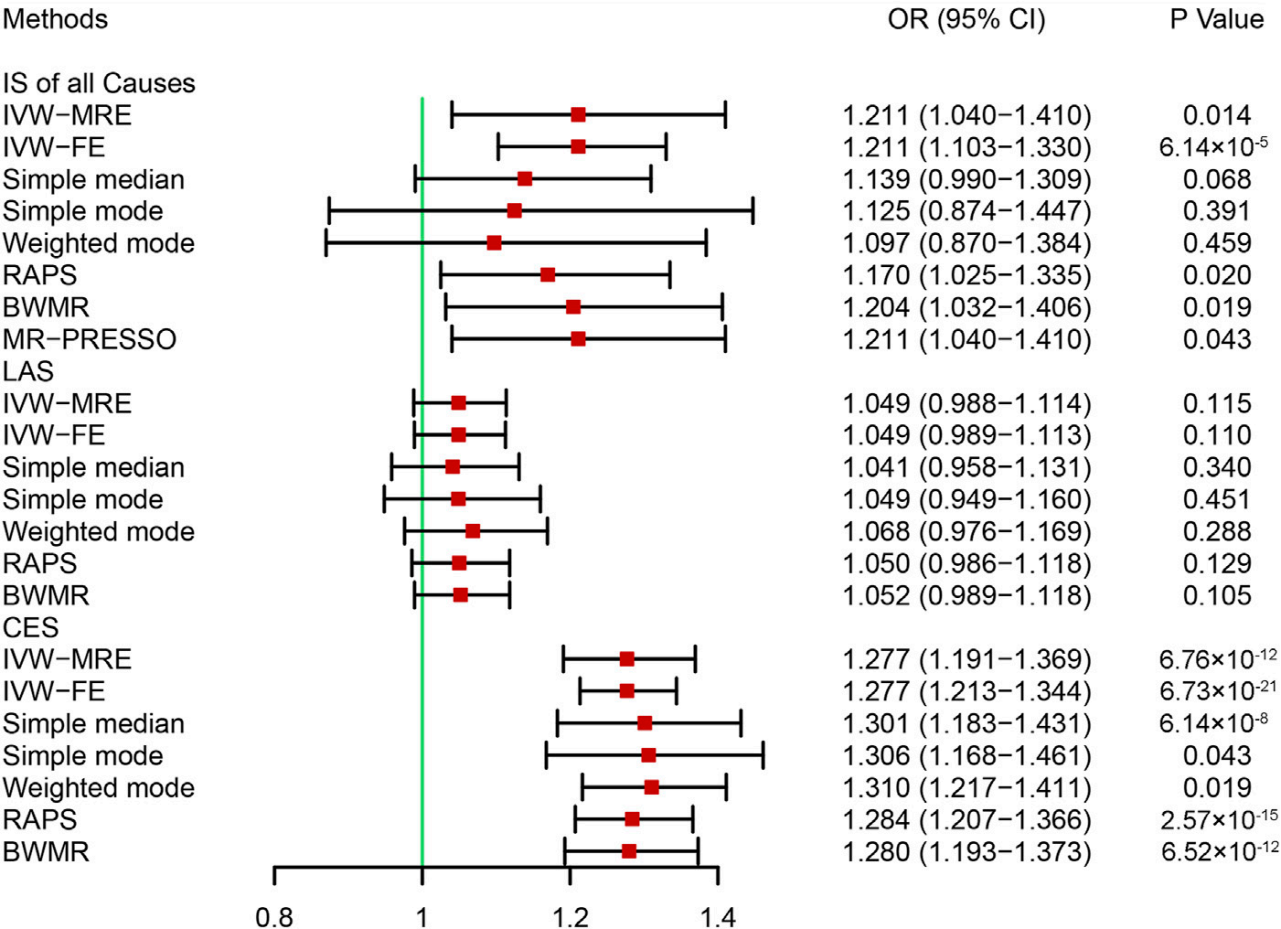
Mendelian randomization analysis of the effect of ischemic stroke on heart failure. BWMR, Bayesian weighted Mendelian randomization; CES, cardioembolic stroke; CI, confidence interval; IS, ischemic stroke; IVW-FE, inverse variance-weighted-fixed effects; IVW-MRE, inverse variance-weighted-multiplicative random effects; LAS: large artery atherosclerosis stroke; MR-PRESSO: Mendelian randomization pleiotropy residual sum and outlier; OR, odds ratio; RAPS, robust adjusted profile score. Bonferroni corrected *p*-value = 0.0017.

A bidirectional MR analysis was performed to evaluate the causal effects of IS on HF. After testing for linkage disequilibrium, 9 SNPs significantly associated with IS of all causes, 4 SNPs significantly associated with LAS, and 4 SNPs significantly associated with CES were identified (Supplementary Table S2). No SNP was identified to be significantly associated with SAS. Finally, 8, 3, and 3 SNPs associated with IS of all causes, LAS, and CES, respectively, were included in the MR analysis (Supplementary Table S2). In the fixed effects IVW analysis, we found causal effects of IS of all causes (OR = 1.211, 95% CI: 1.103–1.330, *p* = 6.14 × 10^−5^) and CES (OR = 1.277, 95% CI: 1.213–1.344, *p* = 6.73 × 10^−21^) on HF (Figure 4). There were no causal effects of LAS on HF (OR = 1.049, 95% CI: 0.989–1.113, *p* = 0.110) (Figure 4). (corrected *p*-value = 0.05/3/1 = 0.017) Sensitivity analysis identified heterogeneity in the analysis between IS of all causes and HF (*p* = 0.016) (Table 2). On the multiplicative random effects model, we found causal effects of IS of all causes on HF (OR = 0.014, 95% CI: 1.103–1.330, *p* = 0.014) (Figure 4). The pleiotropy test indicated no pleiotropy in the analysis, and the results were stable when excluding any single SNP (Table 2 and Supplementary Figure S2).

**FIGURE 5:**
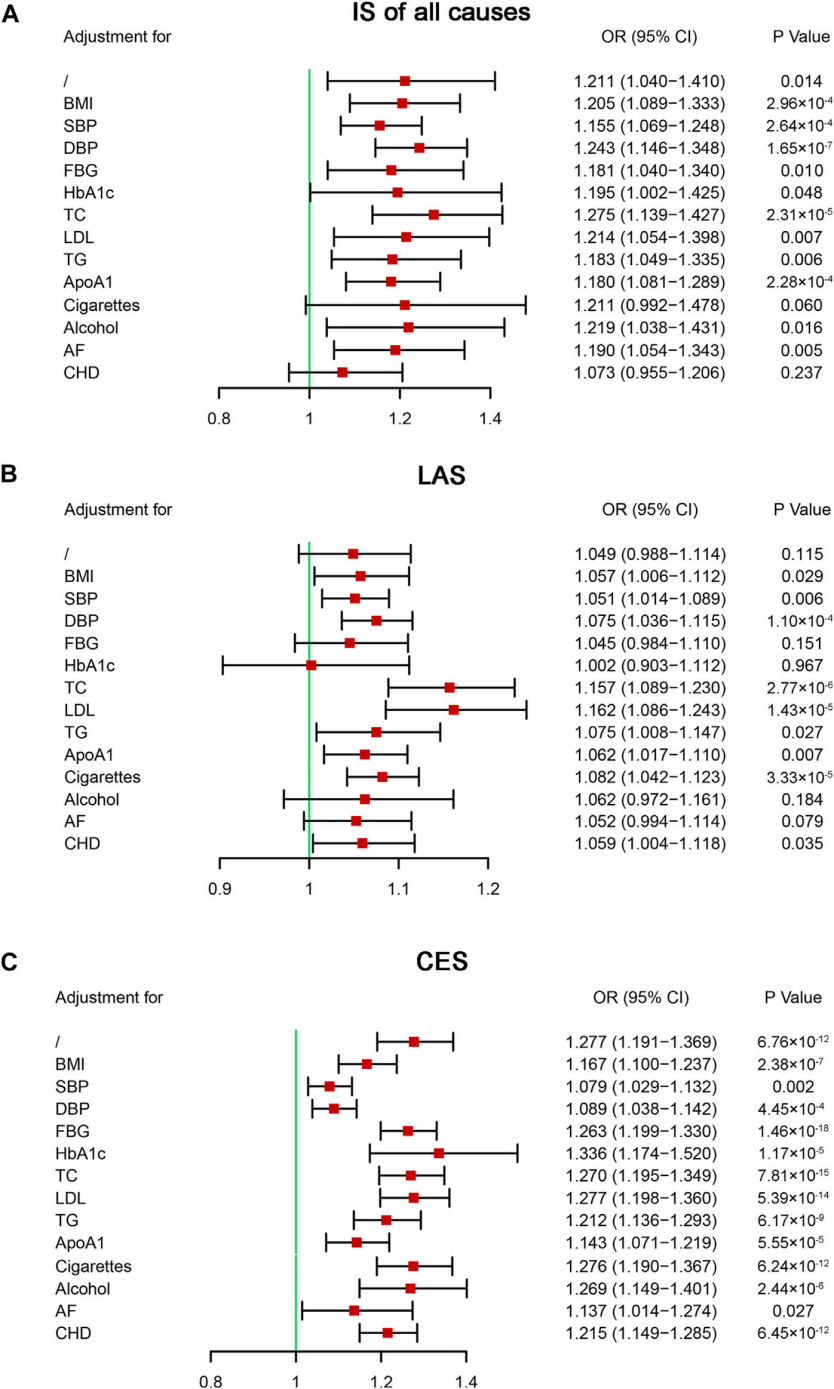
Multivariable Mendelian randomization analysis of the effect of ischemic stroke on heart failure. ApoA1, apolipoprotein A-1; AF, atrial fibrillation; BMI, body mass index; CES, cardioembolic stroke; CHD, coronary heart disease; CI, confidential interval; DBP, diastolic blood pressure; FBG, fasting blood glucose; HbA1c, glycosylated hemoglobin; IS, ischemic stroke; LAS, large artery atherosclerosis stroke; LDL, low-density lipoprotein cholesterol; OR, odds ratio; SBP, systolic blood pressure; TC, total cholesterol; TG, triglyceride; SNP, single nucleotide polymorphism.

Multivariable MR analysis was performed to further evaluate the causal effects of IS on HF. Our analysis indicated that after including CHD, the causal effects of IS of all causes on HF were not statistically significant (OR = 1.073, 95% CI: 0.955–1.206, *p* = 0.237) (Figure 5A). Although two-sample MR analysis indicated no causal effects of LAS on HF, the results of the multivariable MR analysis indicated that some vascular risk factors enhanced the effects of LAS on HF, including BMI, blood pressure, blood lipids, and CHD (Figure 5B). No single vascular risk factor could adjust the causal effect of CES on HF (Figure 5C). Our analysis provided evidence supporting the causal effects of IS of all causes on HF that one unit increase in the genetically predicted log-transformed odds of IS of all causes could increase the risk of HF by approximately 21.1%. Specifically, there was a causal effect of CES on HF, but no causal effect of LAS on HF was identified.

## Discussion

Using MR analysis, our study evaluated the causal link between HF and IS and found that: 1) genetically predicted HF was associated with a higher risk of IS of all causes; 2) subgroup analysis indicated a causal effect of HF on LAS but a suggestive effect of HF on CES; 3) genetically predicted IS of all causes was associated with a higher risk of HF; 4) subgroup analysis indicated a causal effect of CES on HF but no causal link between LAS and HF.

HF, which is characterized by a reduction in ejection fraction and/or an elevation of intracardiac pressure, is the leading cause of hospitalization worldwide (Braunwald, 2013; Kim and Kim, 2018). HF accounts for 2–9% of all stroke cases and predisposes stroke patients to poor prognoses (Solomon et al., 1994; Haeusler et al., 2011). Nevertheless, few studies have focused on the association between HF and IS subtypes, particularly HF and LAS/SAS. The results of this MR study indicated that HF had a causal effect on IS of all causes. Subgroup analysis showed a causal effect of HF on LAS, a suggestive causal effect of HF on CES, and no causal effect of HF on SAS. The neurohormonal mechanism probably plays a role in the relationship between HF and IS (Sokol et al., 2004). With the activation of neurohormonal regulation, particularly the renin-angiotensin-aldosterone system (RAAS), the heart undergoes remodeling and becomes dysfunctional (Udelson and Stevenson, 2016). The activation of RAAS also contributes to endothelial dysfunction and vascular structure damage of the cerebral vessels (Sokol et al., 2004). In addition, HF is accompanied by hypercoagulability, activation of coagulation, inhibition of fibrinolysis, and left ventricular thrombus, which contribute to cerebral thrombogenesis or embolism (Haeusler et al., 2011). Multivariable analysis indicated that CHD and AF adjusted the causal effects of HF on LAS and CES, respectively. AF is one of the most common causes of CES (Mahmood et al., 2014). CHD and IS, particularly LAS, share some common risk factors (Valham et al., 2008). In addition, AF or CHD and HF complicate one other frequently (Ziaeian and Fonarow, 2016; Carlisle et al., 2019). Previous studies revealed that cardioembolism accounted for 82% of stroke etiology in patients with HF and AF, while HF due to CHD was associated with nearly half of LAS cases (Vemmos et al., 2012). Therefore, this study provided evidence supporting the association between HF due to AF and CES as well as HF due to CHD and LAS at the genetic level. In addition, this study also highlights the importance of primary prevention of IS in patients with HF caused by AF or CHD. However, the summary level of the GWAS dataset can hardly eliminate the effect of other confounding factors and avoid the sub-threshold effect of the genetic variants. Therefore, the causal effect of HF on IS, particularly LAS and CES, needs further investigation.

Despite limited attention being paid to the impact of IS on HF, some prior studies have reported that IS is a potential risk factor for HF. Cardiac causes account for 2–6% of deaths in all stroke cases in the first 3 months after IS (Adams et al., 2003). Prosser et al. reported that 19% of all IS patients experienced serious adverse cardiac events in the acute period, including acute myocardial infarction, severe HF, and cardiac death (Prosser et al., 2007). However, few studies have focused on the effects of IS subtypes on HF. The two-sample MR analysis indicated causal effects of IS of all causes on HF, and further subgroup analysis indicated causal effects of CES on HF. However, no causal effects of LAS on HF were identified. The causal effects of CES on HF can be explained by comorbidities with etiologies of HF, like acute myocardial infarction and AF (Ziaeian and Fonarow, 2016). Stroke localization is a potential reason for the association between IS and HF. Previous studies demonstrated that right insular infarction led to a reduction in heart rate variability and a higher occurrence of complex arrhythmias (Tokgözoglu et al., 1999; Colivicchi et al., 2004). In addition, after IS, the blood-brain barrier is disrupted and inflammatory cytokines are released into the systemic circulation, leading to systemic inflammation and immune response (Chen et al., 2017). Thus, our study highlighted the importance of the diagnosis and treatment of CES and its causes, particularly AF, to prevent not only HF, but also recurrent IS.

Our MR analysis identified the causal or suggestive association between HF and LAS/CES at the genetic level. Given the fact that HF and LAS shared several risk factors, the management of risk factors is essential for patients with HF, like lowering the blood pressure, reducing blood lipids, losing weight, and quitting smoking (Barkhudaryan et al., 2021). Furthermore, timely management of etiologies of HF, such as ablation for AF and percutaneous coronary intervention for CHD, are beneficial in preventing both HF itself and IS (Kotecha and Piccini, 2015; Bahit et al., 2018). Regarding the effect of IS on HF, we also showed a causal association between CES and HF by MR analysis, which further emphasized the importance of timely identification of CES and subsequent treatment. Generally, prompt diagnosis of the etiologies of IS and HF is of great importance for the secondary prevention of possible HF or IS.

There were some limitations in our study. First, the definitions of HF were different in the GWAS of HF (Shah et al., 2020). No genetic information about different etiologies (ischemic heart disease, chronic obstructive pulmonary disease, hypertensive heart disease, rheumatic heart disease, etc.) of heart failure is available by the GWAS of HF (Ziaeian and Fonarow, 2016). Therefore, different causes of HF would undoubtedly influence the identification of IVs and their statistical power. The use of summary-level data of GWAS cannot take other risk factors into consideration, thus the effects of other confounders, such as AF, CHD, and hypertension, were inevitable. In addition, other factors like age at disease onset and gender between the GWAS data were another source of bias and were hard to eliminate using summary-level data. Therefore, the genetic variants associated with HF of different etiologies need to be identified in the future. Next, pleiotropy was the main problem in MR analysis, especially when analyzing the association between HF and IS, which shared many common risk factors. In our analysis, we have performed a pleiotropy test, which indicated no existence of pleiotropy. We also performed multivariable MR analysis to avoid pleiotropy. Finally, our MR analysis was based on data from patients of European ancestry and the links between HF and IS are not clear in other ethnicities. The proportions of IS subtypes vary with race and ethnicity (Kim and Kim, 2014). Stroke databases from Western countries suggested cardioembolism was the most common cause of IS in European populations (Stewart et al., 1999; Kolominsky-Rabas et al., 2001). However, in studies conducted in Asian countries (China, Japan, and Pakistan), the prevalence of LAS was higher than CES (Syed et al., 2003; Turin et al., 2010; Tsai et al., 2013). Therefore, unraveling the causal association between HF and different etiologies of IS in other populations, particularly Asian, may provide more information about the common mechanisms or pathways of the two disorders.

Using MR analysis based on summarized data from GWAS, we showed that HF was causally associated with IS of all causes. Subgroup analysis indicated a causal effect of HF on LAS and a suggestive causal effect of HF on CES. Bidirectional MR analysis indicated that IS of all causes was causally associated with HF. Subgroup analysis revealed a causal effect of CES on HF. Our study highlighted the close association between LAS or CES and HF, and the potential causal links need further investigation with genetic information about other ancestries or etiologies of HF.

## Abbreviations

AF, atrial fibrillation; ApoA1, apolipoprotein A-1; BMI, body mass index; BWMR, Bayesian weighted Mendelian randomization; CES, cardioembolic stroke; CHD, coronary heart disease; CI, confidence interval; DBP, diastolic blood pressure; EA: effect allele; EAF: effect allele frequency; FBG, fasting blood glucose; GWAS, genome-wide association study; HbA1c, glycosylated hemoglobin; HF, heart failure; IS, ischemic stroke; IV, instrumental variable; IVW, inverse variance-weighted; IVW-FE, inverse variance-weighted-fixed effects; IVW-MRE, inverse variance-weighted-multiplicative random effects; LAS, large artery atherosclerosis stroke; LDL, low-density lipoprotein cholesterol; MR, Mendelian randomization; MR-PRESSO, Mendelian randomization pleiotropy residual sum and outlier; OA: other allele; OR, odds ratio; RAAS, renin-angiotensin-aldosterone system; RAPS, robust adjusted profile score; SAS, small artery occlusion stroke; SBP, systolic blood pressure; SE, standard error; SNP, single nucleotide polymorphism; TC, total cholesterol; TG, triglyceride.

## Data Availability

The original contributions presented in the study are included in the article/Supplementary Material, further inquiries can be directed to the corresponding authors.
