# Supplementary material for: Heart Failure and Ischemic Stroke: A Bidirectional and Multivariable Mendelian Randomization Study
Source: Front Genet. 2021 Nov 29;12:771044. doi: 10.3389/fgene.2021.771044 (PMC8666512; doi:10.3389/fgene.2021.771044)
Supplement: Supplementary file 1 [file Table1.DOCX]

**Supplementary Table 1. MR results of the effect of heart failure on ischemic stroke**

| SNP | IS of all causes | | |  | LAS | | |  | CES | | |  | SAS | | |
| --- | --- | --- | --- | --- | --- | --- | --- | --- | --- | --- | --- | --- | --- | --- | --- |
|  | β | SE | *p* |  | β | SE | *p* |  | β | SE | *p* |  | β | SE | *p* |
| rs17042102 | 0.102 | 0.015 | 1.99×10^-11^ |  | 0.026 | 0.038 | 0.495 |  | 0.347 | 0.027 | 3.41×10^-37^ |  | 0.003 | 0.036 | 0.927 |
| rs56094641 | 0.011 | 0.010 | 0.305 |  | 0.035 | 0.025 | 0.162 |  | 0.015 | 0.019 | 0.426 |  | 0.017 | 0.023 | 0.456 |
| rs660240 | 0.009 | 0.012 | 0.479 |  | 0.067 | 0.030 | 0.029 |  | 0.013 | 0.024 | 0.588 |  | 0.017 | 0.029 | 0.550 |
| rs1510226 | 0.061 | 0.040 | 0.129 |  | 0.142 | 0.100 | 0.158 |  | 0.052 | 0.083 | 0.530 |  | 0.066 | 0.094 | 0.488 |
| rs17617337 | 0.004 | 0.012 | 0.740 |  | -0.012 | 0.030 | 0.684 |  | 0.053 | 0.023 | 0.020 |  | 0.005 | 0.028 | 0.865 |
| rs11745324 | -0.020 | 0.012 | 0.093 |  | -0.017 | 0.030 | 0.564 |  | -0.076 | 0.024 | 0.001 |  | -0.038 | 0.028 | 0.169 |
| rs55730499 | 0.046 | 0.022 | 0.032 |  | 0.250 | 0.053 | 2.06×10^-6^ |  | 0.041 | 0.043 | 0.338 |  | -0.070 | 0.050 | 0.159 |

CES: cardioembolic stroke; CI: confidential interval; IS: ischemic stroke; LAS: large artery atherosclerosis stroke; MR: Mendelian randomization; OR: odds ratio; SE, standard error; SNP: single nucleotide polymorphism; SAS: small artery occlusion stroke
