# Supplementary figures and images for: Heart Failure and Ischemic Stroke: A Bidirectional and Multivariable Mendelian Randomization Study
Source: Front Genet. 2021 Nov 29;12:771044. doi: 10.3389/fgene.2021.771044 (PMC8666512; doi:10.3389/fgene.2021.771044)

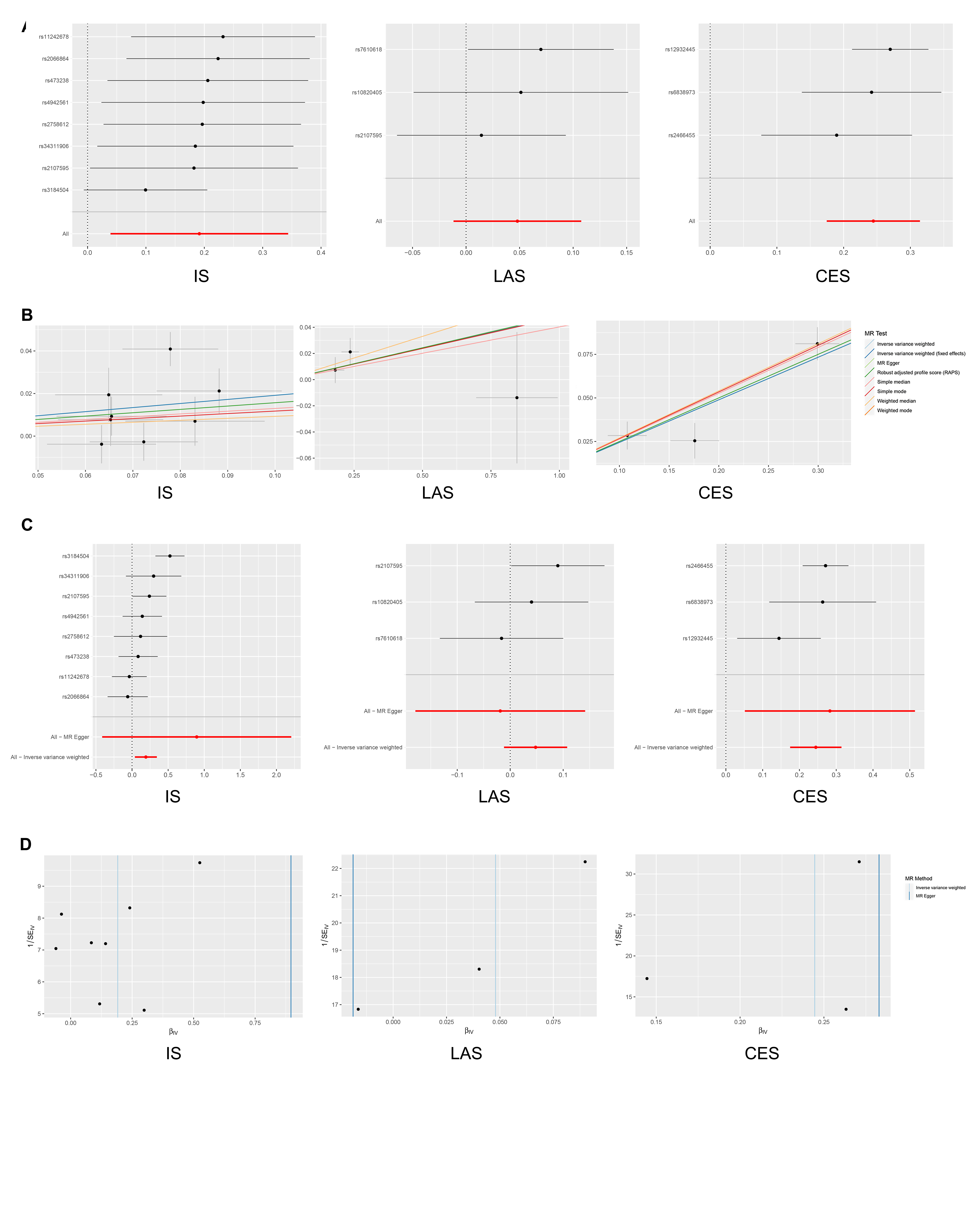

Supplement: Supplementary file 2 [file Image2.TIF]

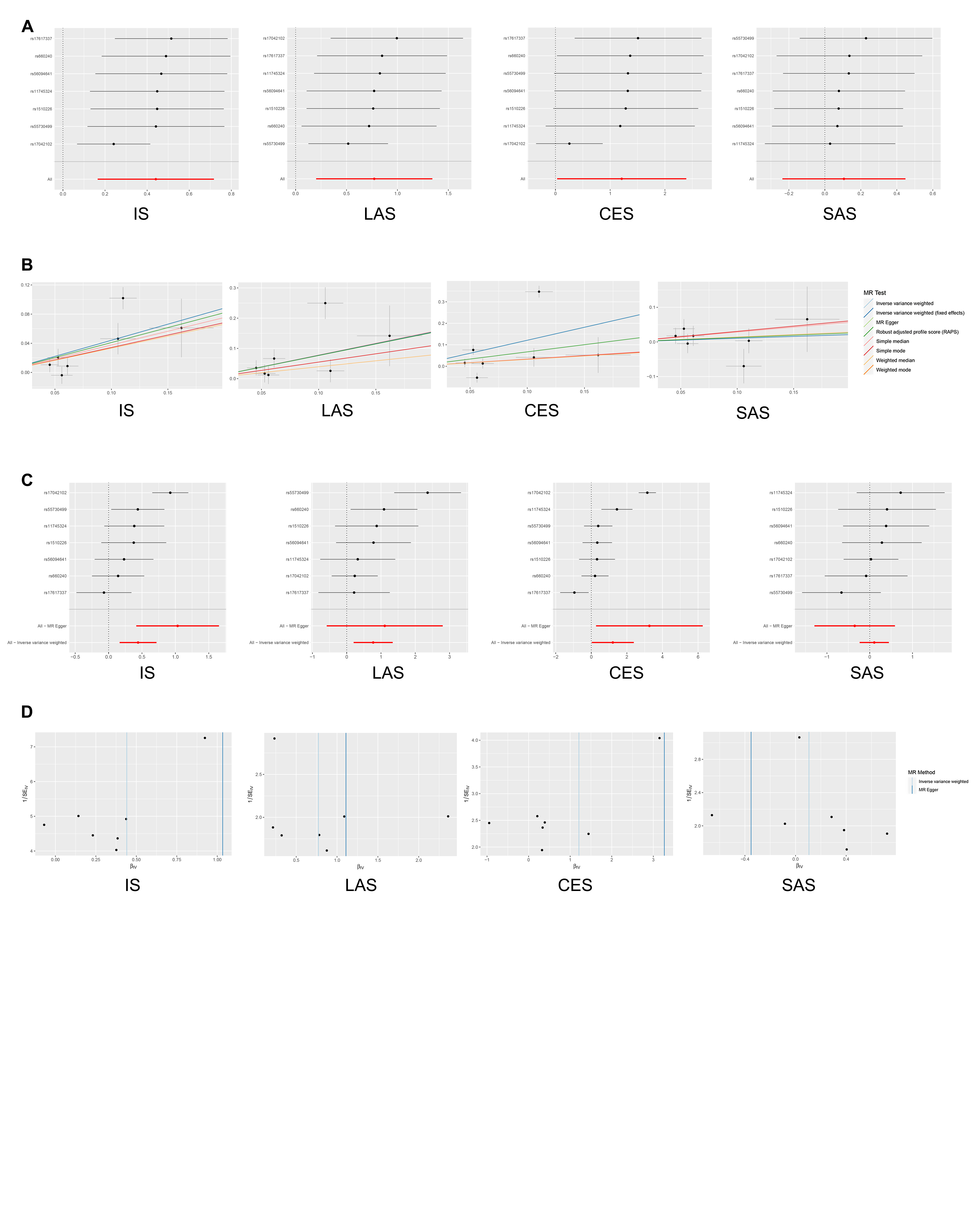

Supplement: Supplementary file 3 [file Image1.TIF]
