# Supplementary material for: Heart Failure and Ischemic Stroke: A Bidirectional and Multivariable Mendelian Randomization Study
Source: Front Genet. 2021 Nov 29;12:771044. doi: 10.3389/fgene.2021.771044 (PMC8666512; doi:10.3389/fgene.2021.771044)
Supplement: Supplementary file 4 [file Table2.DOCX]

**Supplementary Table 2. Included SNPs significantly associated with ischemic stroke**

| SNP | Nearby gene | Ch. | EA | OA | EAF | Exposures | | |  | Heart Failure | | |
| --- | --- | --- | --- | --- | --- | --- | --- | --- | --- | --- | --- | --- |
|  |  |  |  |  |  | β | SE | *p*-value |  | β | SE | *p*-value |
| IS of all causes | | | | | | | | | | | | |
| rs2758612 | *PMF1, PMF1-BGLAP* | 1 | C | T | 0.35 | -0.065 | 0.011 | 3.68×10^-9^ |  | -0.008 | 0.012 | 0.533 |
| rs34311906 | *ANK2* | 4 | C | T | 0.40 | 0.0650 | 0.011 | 1.07×10^-8^ |  | 0.019 | 0.013 | 0.126 |
| rs2634074^*^ | PITX2 | 4 | A | T | 0.79 | -0.094 | 0.012 | 5.90×10^-15^ |  | -0.081 | 0.010 | 3.21×10^-17^ |
| rs2066864 | *FGG* | 4 | A | G | 0.25 | 0.063 | 0.012 | 3.51×10^-8^ |  | -0.004 | 0.009 | 0.674 |
| rs11242678 | *LINC01394, LOC112267978* | 6 | T | C | 0.26 | 0.072 | 0.011 | 2.70×10^-10^ |  | -0.003 | 0.009 | 0.763 |
| rs2107595 | HDAC9–TWIST1 | 7 | A | G | 0.17 | 0.088 | 0.013 | 2.33×10^-11^ |  | 0.021 | 0.011 | 0.046 |
| rs473238 | *WTAPP1* | 11 | C | T | 0.87 | -0.083 | 0.015 | 1.65×10^-8^ |  | -0.007 | 0.012 | 0.544 |
| rs3184504 | *SH2B3* | 12 | C | T | 0.53 | -0.078 | 0.010 | 1.23×10^-14^ |  | -0.041 | 0.008 | 3.32×10^-7^ |
| rs4942561 | *LRCH1* | 13 | T | G | 0.76 | 0.066 | 0.012 | 1.77×10^-8^ |  | 0.009 | 0.009 | 0.302 |
| LAS | | | | | | | | | | | | |
| rs7610618 | TM4SF4–TM4SF1 | 3 | T | C | 0.01 | 0.845 | 0.149 | 1.44×10^-8^ |  | -0.014 | 0.050 | 0.784 |
| rs2107595 | *HDAC9* | 7 | A | G | 0.17 | 0.236 | 0.032 | 1.44×10^-13^ |  | 0.021 | 0.011 | 0.046 |
| rs10820405 | LINC01492 | 9 | A | G | 0.18 | -0.181 | 0.033 | 4.51×10^-8^ |  | -0.007 | 0.010 | 0.463 |
| rs476762^*^ | *MMP3* | 11 | A | T | 0.13 | 0.201 | 0.035 | 1.22×10^-8^ |  | 0.006 | 0.012 | 0.608 |
| CES | | | | | | | | | | | | |
| rs146390073^*^ | *RGS7* | 1 | T | C | 0.02 | 0.669 | 0.120 | 2.20×10^-8^ |  | NA | NA | NA |
| rs2466455 | *-* | 4 | T | C | 0.78 | -0.299 | 0.022 | 2.75×10^-41^ |  | -0.081 | 0.010 | 1.97×10^-17^ |
| rs6838973 | *PITX2* | 4 | T | C | 0.43 | -0.108 | 0.020 | 3.58×10^-8^ |  | -0.028 | 0.008 | 3.67×10^-4^ |
| rs12932445 | *ZFHX3* | 16 | C | T | 0.18 | 0.176 | 0.025 | 6.88×10^-13^ |  | 0.025 | 0.010 | 0.013 |

^*^: not included in the analysis

CES, cardioembolic stroke; Ch., chromosome; EA, effect allele; EAF, effect allele frequency; IS, ischemic stroke; LAS, large artery atherosclerosis stroke; OA, other allele; SE, standard error; SNP, single nucleotide polymorphism
