# Supplementary material for: Heart Failure and Ischemic Stroke: A Bidirectional and Multivariable Mendelian Randomization Study
Source: Front Genet. 2021 Nov 29;12:771044. doi: 10.3389/fgene.2021.771044 (PMC8666512; doi:10.3389/fgene.2021.771044)
Supplement: Supplementary file 5 [file Table3.DOCX]

**Supplementary Table 3. Descriptive information of the studies and datasets included in the analyses**

| **Consortium** | **Phenotype** | **Participants** | **Ancestry** | **Use in this MR study** | **Adjustments*** | **PMID** |
| --- | --- | --- | --- | --- | --- | --- |
| Regeneron Genetics Center | HF | 47,309 cases 930,014 controls | European ancestry | Exposure/Outcome | NA | 31919418 |
| MEGASTROKE | IS and its subtypes (LAS, CES, SAS) | 34,217 cases 406,111 controls | European ancestry | Exposure/Outcome | Age, sex | 29531354 |
| Hoffmann et al. | BMI | 315,347 subjects | European ancestry | Confounder in multivariable MR | Age, sex | 30108127 |
| the International Consortium of Blood Pressure | SBP, DBP | 757,601 subjects | European ancestry | Confounder in multivariable MR | Age, sex, age^2^, BMI | 30224653 |
| Manning et al. | FBG | 58,074 subjects | European ancestry | Confounder in multivariable MR | Age, sex, study-site | 22581228 |
| Prins et al. | HbA1c | 9,436 subjects | European ancestry | Confounder in multivariable MR | NA | 28887542 |
| the Global Lipids Genetics Consortium | TC | 187,365 subjects | European ancestry | Confounder in multivariable MR | Age, sex, age^2^ | 24097068 |
| the Global Lipids Genetics Consortium | LDL | 173,082 subjects | European ancestry | Confounder in multivariable MR | Age, sex, age^2^ | 24097068 |
| the Global Lipids Genetics Consortium | TG | 177,861 subjects | European ancestry | Confounder in multivariable MR | Age, sex, age^2^ | 24097068 |
| UK Biobank | ApoA1 | 393,193 subjects | European ancestry | Confounder in multivariable MR | Age, sex | 32203549 |
| the Tobacco and Genetics Consortium | cigarettes smoked per day | 68,028 subjects | European ancestry | Confounder in multivariable MR | NA | 20418890 |
| Clarke et al. | Alcohol | 112,117 subjects | European ancestry | Confounder in multivariable MR | Age, sex, and four multidimensional scaling components | 28937693 |
| Nielsen et al. | AF | 1,036,836 subjects | European ancestry | Confounder in multivariable MR | Age, sex | 30061737 |
| van der Harst and Verweij | CHD | 547,261 subjects | Most European ancestry (>91%) | Confounder in multivariable MR | Age, sex | 29212778 |

*All GWAS studies have further adjusted for principal components except for HF, HbA1c and cigarettes smoked per day.

Phenotypes: AF: atrial fibrillation; ApoA1: apolipoprotein A-1; BMI: body mass index; CES: cardioembolic stroke; CHD: coronary heart disease; DBP: diastolic blood pressure; FBG: fasting blood glucose; HbA1c: glycosylated hemoglobin; HF: heart failure; IS: ischemic stroke; LAS: large artery atherosclerosis stroke; LDL: low-density lipoprotein cholesterol; SBP: systolic blood pressure; SAS: small artery occlusion stroke; TC: total cholesterol; TG: triglycerides.
